# Opportunities to apply systems engineering to healthcare interprofessional education

**DOI:** 10.3389/fmed.2023.1241041

**Published:** 2023-09-22

**Authors:** Susan Ferreira, Eleanor Phelps, Shannon Abolmaali, Gary Reed, Philip Greilich

**Affiliations:** ^1^Department of Industrial, Manufacturing and Systems Engineering, College of Engineering, University of Texas at Arlington, Arlington, TX, United States; ^2^Office for Quality, Safety and Outcomes Education, University of Texas at Southwestern Medical Center, Dallas, TX, United States; ^3^Department of Anesthesiology and Pain Management, University of Texas Southwestern Medical Center, Dallas, TX, United States

**Keywords:** Interprofessional education, systems engineering, healthcare, state model, framework

## Abstract

In medical settings, interprofessional education (IPE) plays an important role by bringing students from multiple disciplines together to learn how to collaborate effectively and coordinate safe patient care. Yet developing effective IPE is complex, considering that stakeholders from different schools and programs are involved, each with varying curriculum requirements and interests. Given its critical importance and inherent complexity, innovative approaches to address these challenges are needed to effectively develop and sustain effective IPE programs. Systems engineering (SE) combines a lifecycle perspective with established interdisciplinary processes to develop and sustain large complex systems. The need for SE approaches to manage healthcare complexity has been recognized, but the application of SE to IPE programs has been limited. We believe that there is a significant opportunity for IPE programs to benefit from the application of SE. The common themes running through SE and IPE led us to ask if SE can be used to address IPE complexity and achieve desired IPE outcomes. We believe that SE could facilitate further development and sustainability of a recently developed healthcare curriculum. We also propose to use SE to accelerate and manage future IPE curriculum development, while better understanding the states of vital IPE-related components. We discuss a framework that considers transitions of key IPE elements. We believe that use of interdisciplinary SE processes and holistic perspectives and methods such as system thinking will improve the management of system challenges while addressing IPE’s inherent complexity and leading to better patient outcomes and more effective interprofessional collaboration.

## 1. Introduction

Interprofessional education (IPE) brings learners from multiple health professions together to learn how to collaborate and meet objectives such as safety, effectiveness, timeliness, patient-centeredness, efficiency, and equity as presented in the Institute of Medicine’s *Crossing the Quality Chasm* (1). IPE includes occasions when “two or more professions learn with, from, and about each other to improve collaboration and the quality of care and services (2).” Achieving high-reliability in health services and patient-centered care also requires new educational approaches that support clinical transformation toward team-based care. Despite its challenges, developing new IPE curricula that span boundaries among various professions and leverage each profession’s unique expertise to achieve integrated healthcare is a state-of-the-art approach to these transformational objectives.

The Interprofessional Education Collaborative (IPEC) standards established four core competencies for interprofessional collaboration: (1) values/ethics for interprofessional practice; (2) roles and responsibilities; (3) interprofessional communication; and (4) teams and teamwork, and related sub-competencies (3). Unfortunately, a considerable gap in meeting these competencies remains. The barriers to building effective IPE are formidable and overcoming them will require substantial changes in existing attitudes, structure, and processes within academic medical centers (academic medical centers typically integrate patient care with health provider education and research) (4, 5). Successfully addressing these challenges is central to achieving objectives such as the Quintuple Aim (6), which adds health equity to the Quadruple Aim (7) that seeks well-being of the care team as a pre-requisite to the Triple Aim’s (8) objectives of better patient experience, better population health, and lower costs.

Traditionally, systems engineering (SE) considers a full lifecycle, from beginning to end, to define, develop, implement, and sustain complex systems (9). SE is interdisciplinary, involving two or more disciplines (e.g., bodies of knowledge that typically expand over time such as medicine and engineering) (10), and relies on collaboration between stakeholders from different backgrounds working together toward a common set of defined objectives. SE can help manage the inherent complexity of IPE by applying well-established SE processes and concepts such as system thinking to achieve desired IPE outcomes. The need for SE approaches to manage healthcare complexity is already recognized (11–16), as is the need to teach important concepts such as systems thinking to health sciences learners (17). However, application of SE concepts and processes to develop and sustain IPE programs and curricula is currently limited, offering a significant opportunity for these programs to benefit from SE.

We discuss our application of SE concepts and processes, which consider the inherent complexity of IPE, as we developed an IPE program designed to advance teamwork and communication in an academic medical center. We propose the use of SE, specifically a state-based framework, explained in section 4, to accelerate and manage the development and implementation of an IPE curriculum. Using the University of Texas Southwestern Medical Center (UTSW) IPE program as a case study, we discuss the theoretical implications of a tailored SE approach while migrating to a desired future state of a health sciences curriculum.

## 2. Application of systems engineering to interprofessional education

IPE is complex when considering that multiple stakeholders from different schools and programs are involved, each with varying perspectives, curriculum requirements, interests, constraints, and with different learner timelines (e.g., medical students, health professions students, and nursing students). Indeed, an IPE program is a complex system, or even a system of systems (18), requiring different components, relationships between these components, and interactions to successfully create joint curricula, staging activities, and events involving learners and instructors from different professions. Key UTSW IPE requirements included achieving and assessing student learning outcomes, building a cadre of IPE faculty and education scholars, and implementing sustainable organizational changes that will allow the IPE program to evolve as needs and constraints change. SE provides a holistic, methodical, and structured approach to address many IPE challenges.

Many organizations have developed clearly defined processes that they use to specify and develop systems (19–22). Those who create or modify educational curricula can borrow from SE to establish processes and guide their efforts to ensure efficient process development while considering multiple options that will satisfy stakeholder requirements and evolving needs. An interdisciplinary SE approach to IPE enables successful outcomes (9) by using system thinking and integrated processes to solve complex problems while keeping the whole system in perspective over its lifecycle. This lifecycle begins at project conception and continues through defining stakeholder needs and requirements, design, and implementation to delivery of a corresponding solution and sustaining the system (in this case, IPE) through retirement. In addition to basic and clinical sciences, health systems science is an emerging third science specific to healthcare education that was recently adopted and promoted by the American Medical Association (23) and uses systems thinking, which is also core to SE.

The recognized international standard for applying SE to a broad range of systems and products, ISO/IEC/IEEE 15288 (20), provides process descriptions and requirements and identifies four process groups: technical processes, technical management processes, agreement processes, and organizational project-enabling processes. Table 1 identifies the processes associated with each of these groups and presents examples of how some of them could be used to benefit IPE.

**Table 1:** ISO/IEC/IEEE 15288 processes and IPE examples for process use.

Process group	Processes	Examples of process use
Technical	Business or mission analysis Stakeholder needs and requirements definition System requirements definition Architecture definition Design definition System analysis Implementation Integration Verification Transition Validation Operation Maintenance Disposal	Perform stakeholder analysis to determine how to effectively manage stakeholder groups (e.g., learners, facilitators, assessors) associated with IPE; elicit and identify requirements related to educational activities and deliverables including learning outcomes; design IPE curricula to address learning outcomes; perform learner and facilitator assessment. Validate that curriculum meets stakeholder needs through the curriculum lifecycle. Verify that requirements have been met in implemented deliverables. Develop transition plans and use them to transition education deliverables to appropriate stakeholders.
Technical management	Project planning Project assessment and control Decision management Risk management Configuration management Information management Measurement Quality assurance	Use planning, assessment, and control to manage IPE projects. Risk management can be used through the entire curriculum and related activity lifecycles to identify and manage risks, including mitigating high priority risks that could lead to adverse consequences. Perform configuration management to ensure the correct version of education deliverables is used or modified. Assess education related processes, deliverables, and stakeholders to ensure that products meet quality expectations and learner outcomes are achieved.
Agreement	Acquisition Supply	Agreement processes support creation of agreements between organizations to deliver and support products or services. Associated activities can help manage expectations of various stakeholders and internal and external organizations that contribute and participate in developing curricula and learning activities.
Organizational project-enabling	Infrastructure management Portfolio management Human resource management Quality management Knowledge management Life cycle model management	Organizational project-enabling processes apply at an enterprise level and focus on capability, infrastructure, and resources required across many projects. For example, infrastructure management can facilitate resource planning needed across IPE projects (e.g., classrooms, simulation labs, task mannequins and trainers, video equipment) and help manage conflicts and resource shortfalls at the organizational level. Another example is portfolio management that can help assess an IPE project’s contribution to the organization’s strategic plan and a project’s return on investment relative to other projects available for investment.

## 3. Case study: developing an interprofessional healthcare education program at an academic medical center

The University of Texas Southwestern Medical Center (UTSW) has made advancing IPE an institutional priority since 2009 (24). In 2019 UTSW extended this plan based on its alignment with the institution’s 6 year strategic plan and a focus on building further depth in teamwork and communication. These included addressing mandates from the Association of American Medical Colleges (AAMC) for Entrustable Professional Activity (EPA) related to Give or Receive a Patient Handover (EPA #8), Collaborate as a Member of an Interprofessional Team (EPA #9) (25), and the IPE Collaborative’s (IPEC) pillars of teamwork and communication (3). The result was a longitudinal, interprofessional program, Team FIRST, designed to teach core competencies in teamwork to health science students including medical, nursing, and other health profession students (e.g., occupational therapy, physical therapy, pharmacology, physician assistant). Healthcare clinicians must possess teamwork competencies to be effective members of high-reliability teams. The Team FIRST framework identified student learning outcomes linked to ten teamwork competencies that are organized into three domains: communication skills, coordination skills, and handling teamwork challenges. The framework also evaluates the impact of five learning activities by assessing knowledge, skills, and attitudes (KSAs) (26).

This progressive series of five interactive activities includes: introduction to IP teamwork competencies (convergence), introduction to communication competencies, teamwork in the clinical learning environment, just-in-time teamwork clinical series, and using teamwork competencies after graduation. Student training involves four major phases (socialization, application, immersion, and remediation) in simulation- and clinical-based learning environments during their undergraduate education which, for medical students in the US, is the 4 years of medical school after earning their bachelor’s degree. For nurses and health professions students in the US, undergraduate education can include up to a 4 years bachelor’s program after high school depending on the specific profession. Using a triad of students (education), scholars, and (team) scientists, the Team FIRST leadership team built and supports a series of project teams to achieve its learning outcomes that consider important implementation outcomes (e.g., acceptability, appropriateness, and feasibility) (27, 28) prior to executing Team FIRST activities with a high degree of fidelity.

IPE programs have many inherent challenges and barriers (4, 5). Some of the challenges that we faced at UTSW are grouped into four major categories based on perspectives of Team FIRST managers, mentors, and consultants who are authors of this paper (see Table 2). SE processes represented in the ISO/IEC/IEEE 15288 standard and concepts such as systems thinking that could be useful to address these challenges are shown in the third column of the table. SE processes can also be combined with methods from management, human factors, implementation science, and other sciences. Processes such as risk management, configuration management, and other technical management processes apply across the IPE curriculum life cycle. While not a complete list, examples shown in Table 2 highlight complexity as a common pressing challenge that overlays these concerns, and SE can be used to manage complexity.

**Table 2:** UTSW interprofessional education challenge examples & applicable SE processes and approaches.

Challenge category	Challenge	Applicable SE processes or approaches
Curriculum	Achieve consensus on learning outcomes and curriculum requirements from many stakeholders with different interests, opinions, expertise, and experience who come from different organizations.	Stakeholder analysis, business and mission analysis, stakeholder needs and requirements definition, system requirements definition, validation, verification, decision management (e.g., alternative analysis/trade-off analysis), systems thinking.
	Effectively balance priorities related to requirements and constraints to develop an acceptable and feasible curriculum design, implementation, and sustainment strategies.	Decision management, architecture definition, design definition, system analysis, validation, risk management.
Multi-organization scheduling	Manage curriculum schedules for various professions and schedule IPE courses and activities with multiple UTSW and non-UTSW organizations, schools, and department administrators.	Planning, assessment, and control, infrastructure management.
	Manage pre-work and post-activity learner assessments; effective logistics and coordination of pre-work, activity/courses, and post-activity/course assessment of learners and required supporting faculty, staff, evaluators, and other roles.	Measurement, information management, quality assurance, planning, assessment, and control, verification.
Resources	Balance program requirements with constraints such as geographic co-location; fixed facilities, rooms, equipment, and support personnel that limit the number of learners, facilitators and staff that can be scheduled at point in time.	Infrastructure management, planning, assessment, and control.
	Obtain a sufficient quantity of trained and experience facilitators, evaluators, and staff.	Human resource management, knowledge management, portfolio management.
Organization	Organization may not be structurally set up to facilitate development of an evolving IPE program. Many schools were created before recognizing the need for IPE. Organization structures that previously allowed program success may now act as siloes, creating barriers to achieving evolving IPE objectives that require enhanced coordination. For the IPE program at UTSW, AAMC EPA 8 and 9 and IPE competencies must be aligned with the organization’s strategic plan.	Portfolio management, quality management, systems thinking.
	Organizations may have political, relationship or reward barriers that reduce the ability to establish a satisfactory IPE program.	Human resource management, decision management, systems thinking.
	Coordinate complementary curriculum in each School to balance primary educational activities with development of longitudinal teamwork curriculum.	Decision management, planning.
	Alignment and shared control of selected modules within an individual School may need to be modified and enhanced to become interprofessional and achieve desired learning outcomes. Module ownership and contributor shifts may cause disruptions when additional stakeholders seek to broaden the applicability of existing modules.	

## 4. State-based framework for applying system engineering to IPE

**Figure 1:**
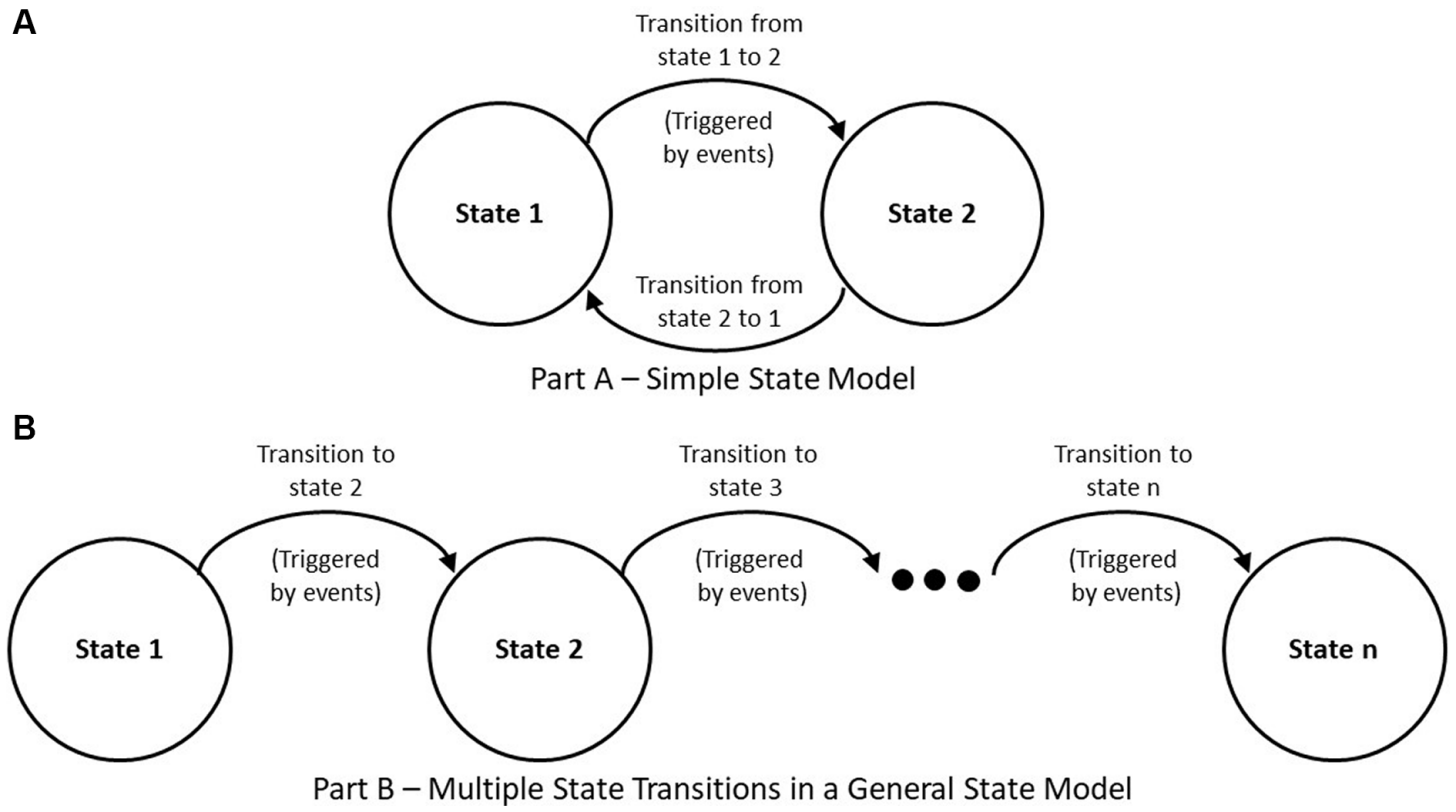
General state-based model. **(A)** Simple state model. **(B)** Multiple state transitions in a general state model.

Based on our experience with Team FIRST and concepts presented in Smartt and Ferreira (29), we propose a general approach to applying SE to IPE based on states of IPE associated entities (or things) as well as processes that are part of the ISO/IEC/IEEE 15288 standard. A framework is a basic conceptual structure (as of ideas) (30). An approach based on an entity’s state considers the state of an entity at a given timepoint, such as a light bulb having two states, “off” or “on.” By turning a switch, we can change the light bulb’s state. Moving from one state to another is based on a decision(s) to initiate an event(s) that triggers an entity to transition from one state to another. The states and transitions are part of a model. See Figure 1A, for a simplified example of transitions within a general state-based model. Note that an entity may also transition back to a previous state. Figure 1B, shows a series of states and transitions with movement between states resulting from a chain of events.

In the Smartt and Ferreira (29) framework, states are defined using four characteristics: organization, environment, process, and product, each of which contribute to IPE development. Each of these categories has multiple attributes. Here, we present an updated framework that considers the four characteristics (organization, environment, process, and product) as distinct entities, each having associated states. Each characteristic group can be subdivided. For example, an organization can be segmented into sub-entities such as projects or teams, each with their own states. An organization may have many teams and many projects, each in a different state at any timepoint, which would allow these entities to also relate to each other in meaningful ways.

**Figure 2:**
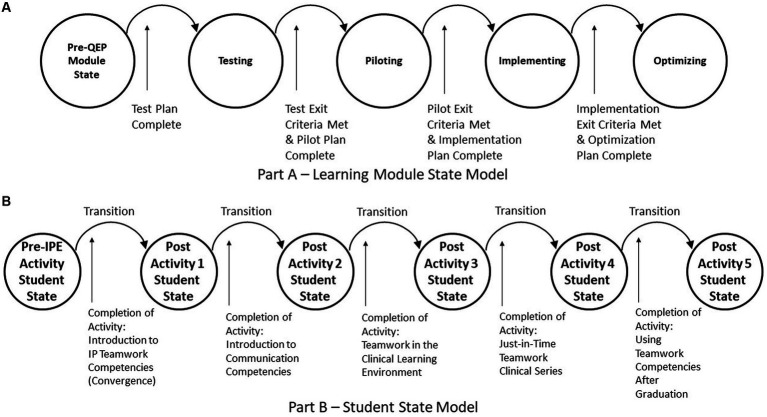
IPE product examples—state models. **(A)** Learning module state model. **(B)** Student state model.

To illustrate use of the state-based framework using the product category as an example, IPE can have different types of products. For example, the UTSW Team FIRST core education products include module curriculum and activities, as well as learner, facilitator, and evaluator assessments. Team FIRST learning modules change their state when exit criteria associated to events are completed. Exit criteria indicate that students, faculty, operational staff, and executive sponsors achieved a sufficient level of acceptability, appropriateness, feasibility, and fidelity to transition to the next state. Figure 2A illustrates the learning module states (testing, piloting, implementing, optimizing that must be passed based on completing the test plan, test exit criteria, pilot plan, and other products).

An educated student is another example of a product of the institution. Changes in a student’s education state(s) occur following exposure to educational materials (e.g., documents, videos, lectures) and by participating in interactive educational activities that lead to specific learning outcomes. Figure 2B illustrates these state changes using the five UTSW IPE and Team FIRST learning modules and associated activities. This figure assumes that modules are taken sequentially, thus completion of each activity would transition the student to the next post activity student state.

## 5. Discussion

The primary goal of Team FIRST was to create and implement learning activities with associated measurement systems and analysis processes that would be able to evaluate Team FIRST learning outcomes. To address this goal, we used the DMADV (define, measure, analyze, design, verify) method, which enables innovative improvements and development of new processes or products (31).

A significant task of the Team FIRST project team was to develop a portfolio of learning activities with behavioral and cognitive performance analysis built on a foundation of continuous quality improvement (QI). These QI principles guided the team to identify low performing sub-activities that could be dropped from the portfolio to improve the performance of other activities. Learner competencies are assessed before and after major activities and survey results are factored into determining low performing sub-activities. Overall, the goal was to identify activities that, when implemented, delivered additional cognitive and behavioral improvements while also improving the satisfaction of learners, instructors, and other stakeholders.

The UTSW Team FIRST project initially applied DMADV components of the ISO/IEC/IEEE 15288 standard processes, which certainly benefited the program however, SE offers a more comprehensive suite of processes than DMADV. While DMADV focuses on improving individual system components, effective IPE requires more comprehensive changes to coordinate and improve multiple components across multiple projects, multiple schools, and the overall organizational environment. For these reasons, the lifecycle perspective and broad array of processes make SE a better approach to address the inherent complexity of IPE. Thus, we are now considering how to best apply SE processes to ongoing IPE efforts, allowing us to achieve desired states for each of the entities involved, e.g., organization(s), team(s), project(s), activity(ies), and learning module(s).

Many organizations already use some features of SE though they may not refer to it by this name. However, to expand the use of SE processes in an organization requires a careful strategy to selectively choose which processes to incorporate and to what level. Doing too much, too quickly, is risky because organizations and individuals need time to understand and respond to emergent concerns when using new processes and methods.

Few studies have been published that describe how to implement SE processes in academic medical centers, health professions institutions, and other healthcare education organizations. Organizations must consider an incremental staged approach that apply selected SE processes so that their utility can be demonstrated and expanded over time. For example, a process might be piloted and evaluated on a single project module and associated activities before broader implementation. As beneficial SE processes are identified, organizations should consider assessing and improving process maturity using models such as Capability Maturity Model Integration (CMMI) (32) to guide further improvements.

While SE can help create, implement, and sustain IPE programs, it will not address all concerns and barriers. SE should be used as an enabler, together with other disciplines and approaches including project management, human factors, team science, and other engineering disciplines along with stakeholders intimately familiar with the needs and concerns of a program and/or organization.

Resources, including dedicated and interested personnel, tools, and equipment, are also needed to introduce the use of SE in an organization. Consultants with SE expertise can help to jumpstart and facilitate progress, but senior leadership and management must also champion these efforts since without them, it will be difficult to obtain the resources, including time and effort, needed to make SE a success.

Standardizing the use of SE within an organization can build consistent practice and scalability however, developing SE capability with its corresponding process improvement will likely require significant time (33). Prior to broad application, new processes need to be carefully planned and introduced to ensure their success, while also considering stakeholder needs, feedback, and lessons learned. Natural resistance to change must also be addressed and managed because the daily business of operating medical and health professions schools places high demand on stakeholders, who are in constant motion with a default mode biased toward past performance.

### 5.1. Challenges and limitations

Several challenges can hinder the successful application of SE to IPE. First, there are insufficient examples and case studies focused on applying SE to health science education. While some health-related cases exist, most SE examples and cases focus on other complex systems such as defense, transportation, communication, and other domains and industries that employ a significant number of systems engineers. The limited availability of systems engineers in health science education is also a significant challenge to applying SE in this setting.

Second, in the absence of examples and exposure to the healthcare domain and concerns, there is a paucity of systems engineers who are sufficiently familiar with the healthcare domain. This is not due to a lack of interest. Indeed, there is interest and growth in healthcare systems courses and programs among systems engineers. The complexity of healthcare, other issues related to social well-being, as well as the need to balance economic and other considerations contribute to challenges that need to be addressed to ensure sustainable outcomes and availability of SE in healthcare domains.

Lastly, the significance of SE and how to deploy systems engineers to address IPE needs to be better recognized within healthcare. As awareness of how to effectively use SE to address problems and create successful healthcare systems grows, more studies will be published about the benefits of SE and increased interest in applying systems engineering will occur.

### 5.2. Conclusion

SE processes can benefit health science IPE. Here we have described the ongoing development of a UTSW IPE program with efforts to apply SE processes to overcome IPE challenges. We discussed a nascent state-based framework with IPE-related examples. This framework will be used to better understand the states of vital IPE-related components during further development and after implementation.

SE can benefit IPE at the level of individual projects all the way to organization levels. In particular, interdisciplinary SE processes will help academic medical centers develop more effective structures and manage requirements and resources, while also helping to address inherent IPE complexity. These impacts can help achieve the Quintuple Aim by allowing teams to more readily address healthcare changes while balancing increasing limitations of financial and other resources at local, regional, and national levels.

The application of systems, industrial, and other engineering principles to healthcare delivery in the United States is long overdue as judged by the absence of sufficient progress in many quality measures over time (34, 35). Appropriate application of SE principles, as illustrated in the program we described, has the potential to reinforce the systems nature of many patient safety issues that plague medicine today. When medical education leaders recognize these issues and begin to apply a team-based, systems focus, our ability to develop a safer care delivery system will greatly improve.

The SE framework presented in this paper provides ideas that can be applied to IPE. Additional research is needed to further evaluate and determine how SE can benefit IPE and other healthcare related concerns, how to perform this evaluation, as well as how SE can be expanded with new approaches to better address healthcare challenges. Our intention is to continue evaluating Team FIRST as a case study and in the application of SE in healthcare education.

## Data availability statement

The original contributions presented in the study are included in the article/supplementary material, further inquiries can be directed to the corresponding author.

## Author contributions

SF conceptualized and led the manuscript development. PG assisted in the conceptualization and draft development. EP and GR developed manuscript sections. SA assisted with draft development. PG and GR provided senior project guidance. All authors contributed to the article and approved the submitted version.

## Funding

The research associated with this paper was funded internally by Team FIRST and the University of Texas Southwestern Medical Center.

## Conflict of interest

The authors declare that the research was conducted in the absence of any commercial or financial relationships that could be construed as a potential conflict of interest.

## Publisher’s note

All claims expressed in this article are solely those of the authors and do not necessarily represent those of their affiliated organizations, or those of the publisher, the editors and the reviewers. Any product that may be evaluated in this article, or claim that may be made by its manufacturer, is not guaranteed or endorsed by the publisher.
